# FLI-1-driven regulation of endothelial cells in human diseases

**DOI:** 10.1186/s12967-024-05546-4

**Published:** 2024-08-06

**Authors:** Lili Zhang, Tingwen Ge, Jiuwei Cui

**Affiliations:** Cancer Center, The First Hospital of Jilin University, No.1 Xinmin Street, Changchun, 130012 China

**Keywords:** FLI-1, Endothelial cells, Systemic sclerosis, Lupus nephritis, Pulmonary hypertension, Tumour

## Abstract

Endothelial cells (ECs) are widely distributed in the human body and play crucial roles in the circulatory and immune systems. ECs dysfunction contributes to the progression of various chronic cardiovascular, renal, and metabolic diseases. As a key transcription factor in ECs, FLI-1 is involved in the differentiation, migration, proliferation, angiogenesis and blood coagulation of ECs. Imbalanced FLI-1 expression in ECs can lead to various diseases. Low FLI-1 expression leads to systemic sclerosis by promoting fibrosis and vascular lesions, to pulmonary arterial hypertension by promoting a local inflammatory state and vascular lesions, and to tumour metastasis by promoting the EndMT process. High FLI-1 expression leads to lupus nephritis by promoting a local inflammatory state. Therefore, FLI-1 in ECs may be a good target for the treatment of the abovementioned diseases. This comprehensive review provides the first overview of FLI-1-mediated regulation of ECs processes, with a focus on its influence on the abovementioned diseases and existing FLI-1-targeted drugs. A better understanding of the role of FLI-1 in ECs may facilitate the design of more effective targeted therapies for clinical applications, particularly for tumour treatment.

## Introduction

Endothelial cells (ECs) are highly specialized cells that are located in the heart, blood vessels and lymph vessels [1]. On the basis of their structure and distribution, ECs can be divided into vascular ECs and nonvascular ECs, among which vascular ECs are present in arteries, arterioles, arterial capillaries, capillaries, venous capillaries, postcapillary venules and veins [2]. In the human body, ECs not only deliver oxygen and nutrients, regulate blood flow and assist in haemostasis but also play a key role in regulating immune cell transport and maintaining tissue homeostasis, constituting an important part of the circulatory system and immune system [1]. ECs dysfunction can lead to the progression of a variety of chronic cardiovascular, renal and metabolic diseases [3].

FLI-1, also known as Friend leukaemia virus integration 1 [4], is a transcription factor in the E26 transformation-specific (ETS) gene family [5]. The FLI-1 gene is located on mouse chromosome 9 and on human chromosome 11q23 and contains three functional domains: an FLI-1-specific (FLS, aa 205–292) domain, a carboxy-terminal transcriptional activation (CTA, aa 402–452) domain, and an amino terminal transcriptional activation (ATA) domain [6]. As a transcription factor, FLI-1 not only inhibits transcription or activates transcriptional gene expression by specifically binding promoters or enhancers but also plays a regulatory role at the transcriptional level through protein interactions [6]. Under physiological conditions, FLI-1 is mainly expressed in ECs, haematopoietic stem cells, fibroblasts, immune cells and various blood cells and regulates their differentiation, migration, proliferation and other biological functions [7]. For example, FLI-1 is involved in immune cells function by controlling proliferation, activation, migration and regulating cytokine and chemokine production; regulating the differentiation of stem cells into mature blood cells of different lineages; and regulating the transcription of genes related to angiogenesis to control ECs survival and vascular development [8]. The abnormal expression of FLI-1 can affects normal physiological processes in cells and lead to the occurrence and development of several diseases. Overexpressing FLI-1 in haematopoietic progenitor cells inhibits CD4( +) T cells differentiation and enhances CD8( +) T cells development, which may eventually lead to pre-T cell lymphoblastic leukaemia/lymphoma in transgenic mice; downregulating the expression of FLI-1 in fibroblasts may lead to SSc; and a lack of FLI-1 in megakaryocytic progenitor cells results in a defect in megakaryopoiesis, which can lead to Jacobsen or Paris-Trousseau syndrome [9].

According to the literature, FLI-1 also as a key transcription factor is involved in biological functions of ECs. The abnormal expression of FLI-1 in ECs can lead to the occurrence and development of systemic sclerosis (SSc), lupus nephritis (LN), pulmonary hypertension (PAH) and tumours. However, the regulatory mechanisms of FLI-1 in ECs are not fully understood. Therefore, this comprehensive review provides the first overview of FLI-1-mediated regulation of ECs processes, including differentiation, proliferation, migration, angiogenesis and blood coagulation. Additionally, we analyse the influence of FLI-1 dysfunction in ECs on the abovementioned diseases. Finally, we provide insight into the classifications, mechanisms and potential advantages of FLI-1-targeted drugs. An improved understanding of FLI-1 in ECs may help in the design of more effective targeted therapies, especially for clinical application in the treatment of tumour patients.

## Materials and methods

We synthesized the results reported in the literature obtained by searching digital databases, consulting authoritative texts and conducting manual searches of references included with the retrieved literature. We retrieved about 1420 articles, screened them by topic, summarized and refined the results of the selected literature, and then compiled the review. The specific search strategy is detailed in the search strategy summary in Table 1.

**Table 1:** Search strategy summary

Items	Specification
Date of search	March 29, 2024
Databases and other sources searched	Electronic searches of PubMed, manual searches of references included with retrieved literature, and consultation of authoritative texts
Search terms used	FLI-1 AND (Endothelial cells OR Systemic sclerosis OR Lupus nephritis OR Pulmonary arterial hypertension OR Tumour OR Cancer OR Inhibitors OR Activators OR Nanomaterials OR Antibody drug conjugates)
Timeframe	January 1990-March 29, 2024
Number of articles	1420
Inclusion and exclusion criteria	The articles had to describe the role of FLI-1 in endothelial cells or describe FLI-1-targeted drugs. No language restrictions were imposed
Selection process	Two authors (L.L. and T.W.) reviewed independently and in duplicate retrieved articles for inclusion based on title and abstract and reviewed the full text of relevant articles. Disagreement during the review process was resolved by consensus through involvement of a third review author (J.W.)

## Regulatory effect of FLI-1 on ECs

### FLI-1 regulates the differentiation of ECs

Most ECs are derived from pluripotent stem cells (PSCs), and the differentiation process can be divided into two stages (S1 and S2). In the S1 stage, PSCs differentiate into mesodermal progenitor cells (MPCs), a process mainly regulated by the WNT signalling pathway. In the S2 stage, MPCs differentiate into ECs through vascular endothelial growth factor (VEGF) signal transduction [10]. Studies have shown that ECs differentiation is also co-regulated by many other signalling pathways and transcription factors, such as the bone morphogenetic protein (BMP) signalling pathway, the Notch signalling pathway, angiopoietin-1 and tyrosine kinases, and transforming growth factor-β (TGF-β) [11].

**Fig. 1:**
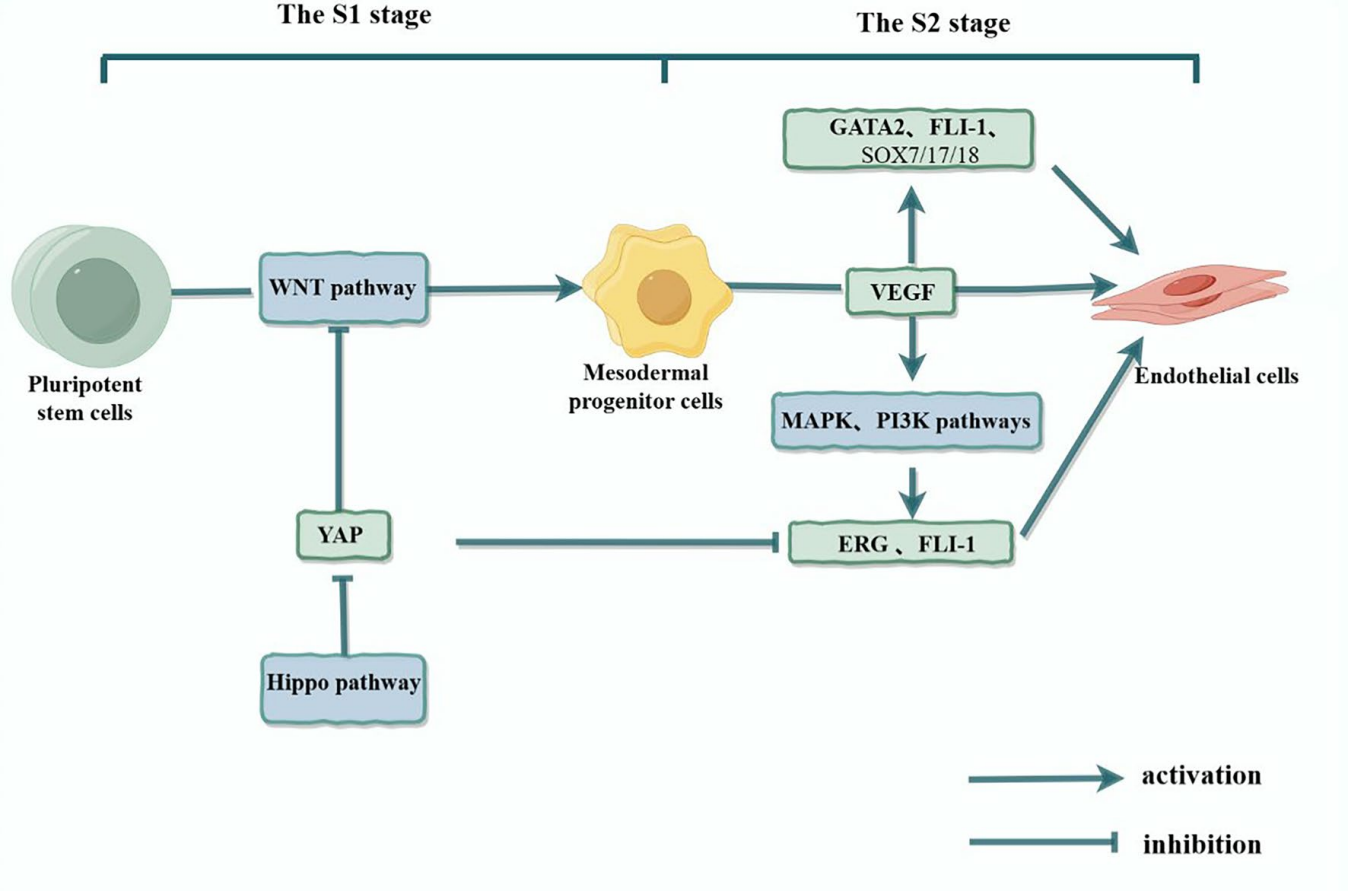
Schematic diagram of the involvement of FLI-1 in the regulation of endothelial cells differentiation. The differentiation process of endothelial cells can be divided into two stages (S1 and S2). FLI-1 is involved mainly in the S2 stage: (1) as a downstream target of the MAPK and PI3K pathways; (2) as a downstream target of the Hippo pathway; and (3) in combination with other transcription factors

FLI-1 is involved mainly in the S2 phase of ECs differentiation, as follows: (1) as a downstream target of the MAPK and PI3K pathways; (2) as a downstream target of the Hippo pathway; and (3) in combination with other transcription factors (Fig. 1). On the basis that VEGF can activate the MAPK and PI3K pathways, Aja Harding et al. applied MAPK and PI3K pathway inhibitors to MPCs in vitro growth medium and found that the expression levels of the EST family transcription factors ERG and FLI-1 were decreased. The expression of ECs markers, such as VE-cadherin, CD31 and von Willebrand factor (VWF), was significantly reduced, indicating that ERG and FLI-1 most likely mediate the influence of the MAPK and PI3K pathways on the differentiation of ECs [12]. Similarly, Quan et al. through the knockdown and overexpression of YES-associated protein (YAP) in vitro growth medium, reported that the Hippo pathway plays a role in two stages of ECs differentiation by inhibiting YAP: YAP not only inhibits MPCs differentiation by blocking the WNT signalling pathway but also affects ECs differentiation by inhibiting key transcription factors, such as FLI-1, SOX17, and ERG [13]. As a YAP inhibitor, degenerate family member 4 can inhibit YAP and upregulate FLI-1 expression to promote ECs differentiation [14]. In addition, Kanki et al. used genomic and epigenome methods to analyse ECs differentiation and reported that GATA2, FLI-1 and SOX7/17/18 are combined transcription factors involved in ECs differentiation; these transcription factors can facilitate changes in histone modifications from H3K27me3 to H3K4me3 after VEGF stimulation throughout the mesoderm period, promoting ECs differentiation [15]. The combined action of these transcription factors not only promotes the differentiation of ECs but also prevents the differentiation of PSCs into other cell types. For example, GATA2 and FLI-1 inhibit the differentiation of PSCs into blood cells or vascular smooth muscle cells; SOX18 and GATA2 inhibit the differentiation of PSCs into cardiomyocytes; and SOX7 inhibits the differentiation of PSCs into mesenchymal cells [15].

Due to the important role of FLI-1 in ECs differentiation, the regulation of FLI-1 expression is currently included as an approach to induce efficient ECs differentiation. For example, in vitro cell culture medium, the inhibition of TGF-β and constitutive expression of FLI-1 and ERG can transform amniotic cells into mature ECs [16]; the simultaneous overexpression of FLI-1 and activation of protein kinase C (PKC) can induce the rapid and effective differentiation of embryonic stem cells into ECs within 3 days [17]; the overexpression of three key ETS transcription factors, ETV2, FLI-1, and ERG, can reprogramme induced brain microvascular endothelial cells (iBMECs) into authentic ECs [18].

### FLI-1 regulates the proliferation and migration of ECs

ECs proliferation and migration are crucial for neovascularization, and these processes can also be regulated by VEGF, Notch, BMP and other signalling pathways [11]. FLI-1 affects the proliferation and migration of ECs by regulating the expression of genes related to proliferation and migration.

Some studies have shown that FLI-1 overexpression promotes ECs proliferation and migration. Jiang et al. added CXCL4 plasma (with or without neutralizing antibody against CXCL4) to the culture medium of ECs, and found that CXCL4 directly inhibited the proliferation and migration of ECs by downregulating the expression of FLI-1 [19]. In a zebrafish model, Matrone et al. inoculated ECs with differentially expressed FLI-1 into the medium, and found that among the proliferating ECs, the number of ECs with high FLI-1 expression was significantly greater than that of ECs with low FLI-1 expression (45% vs. 5%) [20]. This proliferative effect was subsequently found to be due to FLI-1-mediated transcriptional suppression of the expression of genes associated with anti-proliferative cell responses, such as FOXO3A [21]. In terms of migration, FLI-1 is located upstream of zinc finger domain transcriptional regulator (lmo2), promoting its expression [22]. Lmo2 promotes ECs migration by regulating sphingosine kinase [23].

Some studies have shown that FLI-1 inhibits ECs proliferation and migration. In ECs with normal FLI-1 expression and ECs in which FLI-1 is knocked out, Tetsuo et al. reported that the former showed a low proliferation rate in a BrdU assay and a low migration potential in a scratch assay and transmigration assay [24]. Subsequently, Marden et al. reported that FLI-1 could inhibit the expression of oncostatin M receptor (OSMRβ) in ECs, with ECs exhibiting obvious proliferation and migration after OSM stimulation [25]. Similarly, FLI-1 can inhibit the expression of an adipokine (chemerin) the promotes cell proliferation [26]. In terms of migration, FLI-1 inhibits the expression of αVβ3 integrin, which promotes ECs migration through interaction with VEGFR2 [24].

### FLI-1 regulates angiogenesis

Angiogenesis refers to the process of budding from the existing vascular system to form new blood vessels [13]. Previous studies have shown that the targeted deletion of FLI-1 in mice leads to foetal mortality, possibly due to abnormal bleeding caused by vascular integrity defects during vascular development [27], indicating that FLI-1 may play a key role in vascular development. The functional region of FLI-1 in ECs has been screened by ChIP analysis, and the FLI-1 peak was found to be highly enriched in the proximal region of genes related to vascular morphogenesis and angiogenesis [28], suggesting that FLI-1 affects angiogenesis mainly by regulating the expression of genes related to vascular morphogenesis and vascular homeostasis.

Using loss- and gain-of-function strategies and a series of molecular techniques, Abedin et al. reported that in the process of mouse embryonic blood vessel morphogenesis, the synergistic transcriptional activity of Etv2 and FLI-1 regulated blood vessel morphology at different stages. In early pregnancy, Etv2 can initiate embryonic angiogenesis by binding to ETS binding sites in the promoter region of the FLI-1 gene to control FLI-1 expression. When the expression of the Etv2 gene is stopped in the second trimester, FLI-1 can autonomously regulate its own expression and the expression of Etv2 target genes (such as ERG, Cdh5, and Tie2) to promote vascular morphogenesis in the second and third trimesters [29]. Recent studies have also shown that FLI-1 in middle and late pregnancy can promote endothelial gene expression and inhibit the expression of myogenic-related genes (such as Myf5), resulting in vascular morphogenesis [30].

In addition to regulating vascular morphogenesis, Asano et al. reported that compared with wild-type mice, mice with EC-specific FLI-1 deletion (FLI-1CKO) exhibited abnormal expression of genes related to vascular stability; for example, the expression of VE-cadherin, platelet endothelial cell adhesion molecule-1, type IV collagen, platelet-derived growth factor B and other proteins decreased, and the expression of matrix metalloproteinase 9 protein significantly increased. According to ChIP analysis, these genes are directly regulated by FLI-1 [31]. Zeng et al. showed that FLI-1 knockdown significantly reduced VEGFA expression, further confirming the important role of FLI-1 in maintaining vascular homeostasis [32].

### FLI-1 regulates blood coagulation

Blood coagulation is an important part of haemostasis. ECs play a key role in blood coagulation. When activated, ECs can interact with platelets, red blood cells and coagulation factors to promote blood coagulation [33]. FLI-1 in ECs mainly regulates the expression of coagulation factors and plasma proteins and affects blood coagulation.

In mice with combined deficiency FLI-1 and ERG in ECs, Gomez-Salinero et al. observed the decreased expression of vascular components of anticoagulation and antithrombotic signalling pathways, such as Nos3, Thbd and Tfpi. Moreover, the expression of procoagulant and prothrombotic response genes, such as Par1 protease activating receptor 1 (F2r) and platelet reactive protein 1 (Thbs1), increased, indicating that FLI-1 is involved in blood coagulation [34]. The endothelial protein C receptor (EPCR) is mainly expressed on ECs and binds and activates protein C before releasing it. Protein C interacts with the phospholipid membrane of ECs to inactivate factor Va and factor VIIIa [35], which play key roles in the regulation of the coagulation system. Saigusa et al. reported that FLI-1 occupies the promoter region of the EPCR coding sequence (PROCR) and that *FLI-1* gene silencing leads to the inhibition of EPCR expression [36] and thus promotes blood coagulation. However, Ng et al. showed that FLI-1 knockdown in ECs can cause bleeding. VWF is a key coagulation protein mainly derived from ECs that can not only mediate the adhesion of platelets to vascular injury sites but also stabilize factor VIII [37]. FLI-1 transcription promotes VWF promoter activity, and VWF mRNA expression decreases after FLI-1 knockdown [37].

## Diseases caused by abnormal FLI-1 expression in ECs

### The mechanisms of action of FLI-1 in the occurrence and development of SSc

SSc is a complex autoimmune disease with characteristic clinical manifestations that include microvascular lesions, chronic inflammation, and excessive fibrosis of the skin and internal organs (such as the lungs, heart, and gastrointestinal tract) [38]. At present, the pathogenesis of SSc has not been fully elucidated [38]. The known mechanisms include the dysfunction of ECs and immune cells, as well as the abnormal secretion of inflammatory and fibrotic cytokines [39]. Low FLI-1 expression in fibroblasts and ECs plays an important role in the pathogenesis of SSc [24]. In a previous study, the vascular changes in FLI-1 CKO mice were similar to the changes observed in SSc patients, indicating that FLI-1 deficiency in ECs may be a key inducible factor for SSc [31].

FLI-1 levels are regulated by a variety of mechanisms, including transcriptional regulation, posttranslational modification, and epigenetic modification [18], but most regulation occurs at the protein level [40]. Studies have shown that the lysosomal enzyme cathepsin B (CTSB) can degrade *FLI-1* [40]. The chemokine CXCL4 can downregulate the expression of *FLI-1* through the c-Abl pathway [19]. Other inflammatory mediators, including IFN-α, TLR ligands, TGF-β and endothelin ET-1, also reduce *FLI-1* levels in ECs [25].

**Fig. 2:**
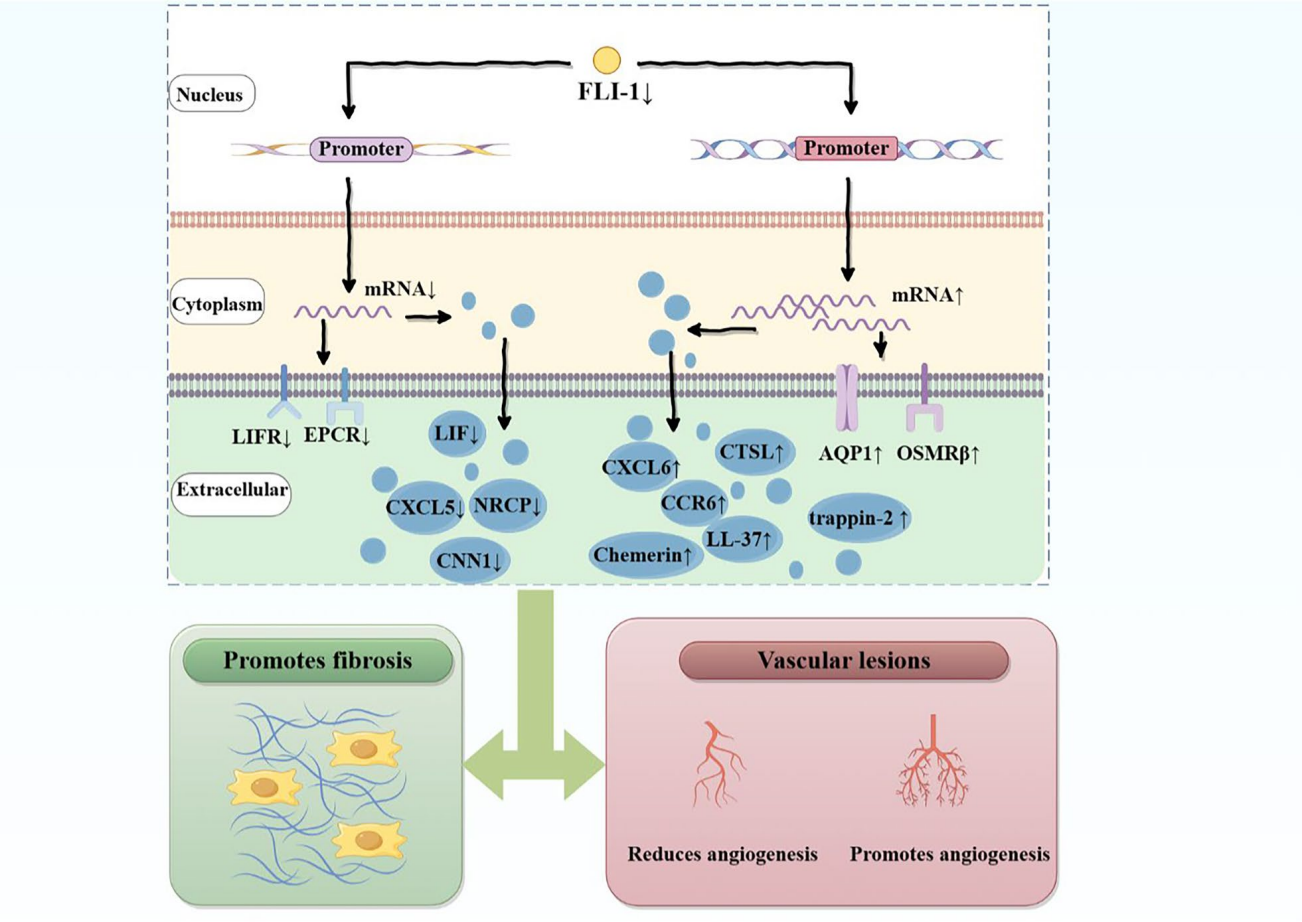
Schematic diagram of the effect of endothelial cell FLI-1 on systemic sclerosis. Low expression of FLI-1 in endothelial cells can result in upregulation of CTSL, trappin-2, adipsin, LL-37, CCR6, CXCL6, OSMRβ and AQP1 and downregulation the expression of EPCR, CXCL5, CNN1, LIF, LIFR and NRCP. Abnormalities in these factors cooperate to promote fibrosis and vascular lesions

FLI-1 in ECs mainly affects tissue fibrosis and angiogenesis by regulating the expression of various cytokines in SSc (Fig. 2). Chemokines are proteins that can recruit inflammatory cells to specific tissues and organs [41]. A variety of CXC chemokines, such as CXCL4 [19], CXCL5 [42], CXCL6 [43], CXCL12 [44], CXCL13 [45] and CXCL14 [46], have been shown to be associated with the progression of SSc. CXCL5 and CXCL6 have been shown to be directly regulated by FLI-1 in ECs. FLI-1 deficiency can decrease the expression of CXCL5 [42] and upregulate the expression of CXCL6 [43] in ECs, resulting in the development of finger ulcers and excessive fibrosis and accelerating the development of SSc. Recent studies have shown that the chemokine CCR6 is also involved in the development of SSc and is regulated by FLI-1. FLI-1 deficiency can increase CCR6 expression in ECs, further aggravating SSc vascular disease [41].

In addition to chemokines, other cytokines in ECs are also regulated by FLI-1. OSM not only plays a role in ECs migration and proliferation but also plays a certain role in promoting fibrotic gene expression. The downregulation of FLI-1 expression can promotes high OSMRβ expression in ECs and thus promote fibrosis in various organs caused by OSM [25]. Aquaporin-1 (AQP1) is a cell membrane protein that is highly expressed in ECs and fibroblasts, and its degree of expression is proportional to tissue fibrosis. Yamashita et al. reported that the promoter of the AQP1 gene in SSc ECs is directly regulated by FLI-1 and that a lack of FLI-1 enhances the expression of AQP1 mRNA and promotes fibrosis [47]. In addition, FLI-1 can also regulate the expression of leukaemia inhibitory factor (LIF) and its receptor, trappin-2, adipsin, antimicrobial peptide LL-37, cathepsin L (CTSL), EPCR, neuropilin 1 (NRP1), chemerin and calponin 1 (CNN1) in ECs, further affecting SSc lesions[26, 36, 48–54] (Table 2).

**Table 2:** List of cytokines involved in systemic sclerosis regulated by FLI-1 in ECs and their mechanisms of action

Cytokine	Regulatory mechanism of FLI-1	Effect on SSc	References
CXCL5	Binds to the CXCL5 promoter	Vascular lesions	[42]
CXCL6	Binds to the CXCL6 promoter	Vascular lesions and fibrosis promotion	[43]
CCR6	Binds to the CCR6 promoter	Vascular lesions	[41]
OSM	Regulates OSMR expression	Fibrosis promotion	[25]
AQP1	Binds to the AQP1 promoter	Vascular lesions and fibrosis promotion	[47]
LIF	Binds the LIF and LIFR promoters	Vascular lesions	[48]
Trappin-2	Binds the PI3 promoter	Vascular lesions	[49]
Adipsin	Binds the adipsin promoter	Vascular lesions	[50]
LL-37	Binds the CAMP promoter	Vascular lesions and fibrosis promotion	[51]
CTSL	Binds the CTSL promoter	Vascular lesions	[52]
EPCR	Binds the PROCR promoter	Vascular lesions and fibrosis promotion	[36]
NRP1	Binds the NRP1 promoter	Vascular lesions	[53]
Chemerin	Binds the chemerin promoter	Vascular lesions	[26]
CNN1	Binds the CNN1 promoter	Vascular lesions	[54]

*ECs* endothelial cells, *SSc* systemic sclerosis, *OSM* oncostatin M, *OSMR* oncostatin M receptor, *AQP1* aquaporin-1, *LIF* leukaemia inhibitory factor, *LIFR* leukaemia inhibitory factor receptor, *CAMP* encoded LL-37 gene, *CTSL* cathepsin L, *EPCR* endothelial protein C receptor, *PROCR* also called endothelial protein C receptor, *NRP1* neuropilin 1, *CNN1* calponin 1

### The mechanisms of action of FLI-1 in the occurrence and development of LN

Systemic lupus erythematosus (SLE) is a systemic autoimmune disease that involves multiple target organs and causes multisystem immune damage. LN is a common serious complication in SLE patients, and its clinical manifestations are variable, ranging from asymptomatic proteinuria to overt proteinuria, nephrotic syndrome or nephritic syndrome, and end-stage renal disease [55]. The pathogenesis of LN involves a variety of intrarenal and extrarenal pathogenic mechanisms. The intrarenal mechanisms include antibody binding to intrarenal autoantigens and local complement and Fc receptor (FcR) activation. Extrarenal mechanisms include different combinations of genetic variants in patients [56]. The overexpression of FLI-1 in healthy mice leads to lupus-like lesions [57], while low FLI-1 expression significantly reduces pathological changes in the kidneys in mouse models of lupus, thereby prolonging their survival [58]. Suzuki et al. reported that FLI-1 was expressed mainly in renal ECs [58], but it was not clear how FLI-1 was overexpressed in LN ECs.

**Fig. 3:**
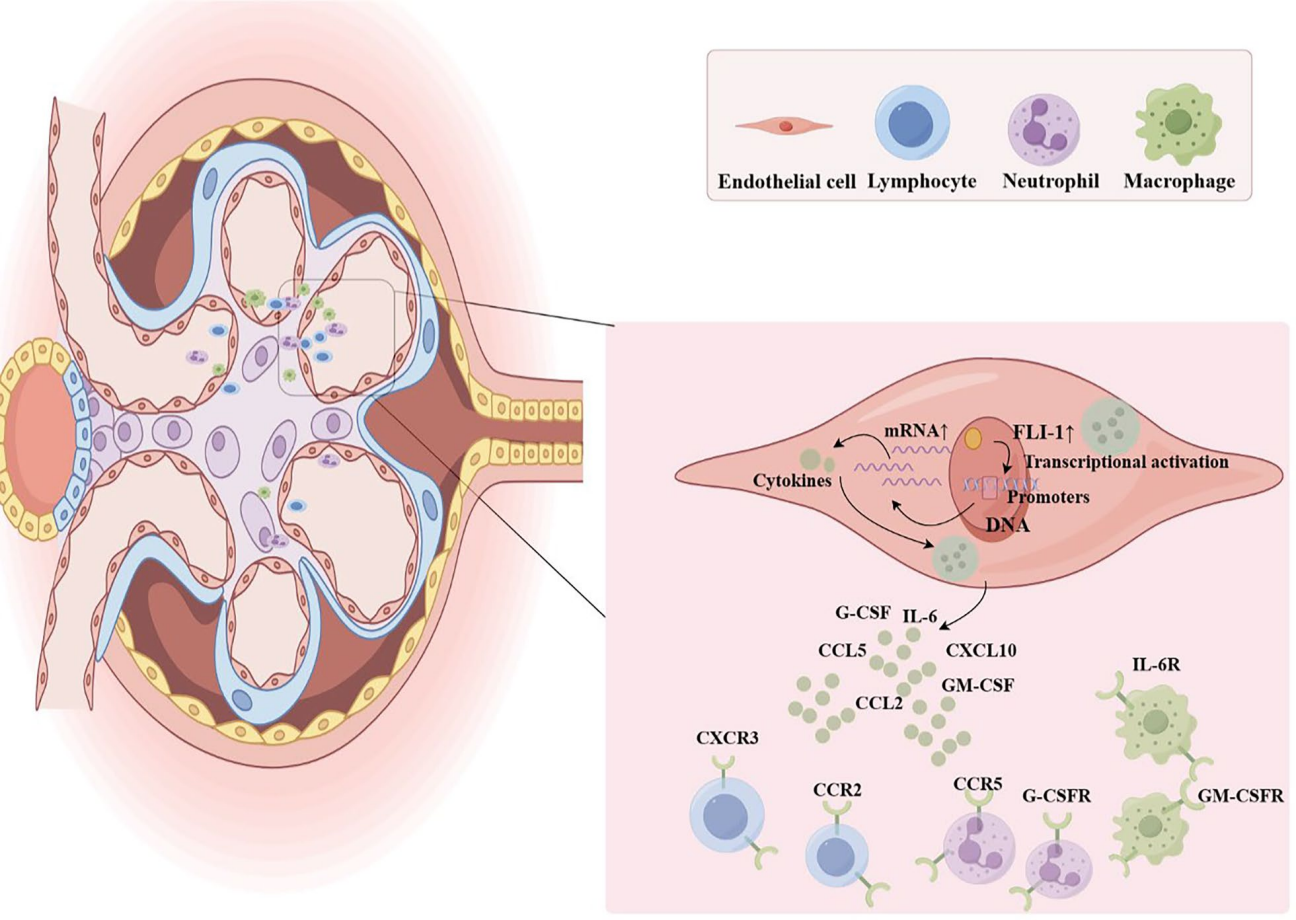
Schematic diagram of the role of endothelial cell FLI-1 in lupus nephritis. High expression of FLI-1 in endothelial cell binds to the promoters of inflammatory factors, such as CXCL10, IL-6, G-CSF, GM-CSF, CCL2, and CCL5, upregulating their expression to influence the infiltration of inflammatory cells into the kidney

FLI-1 in ECs plays a role in LN by influencing the infiltration of inflammatory cells into the kidney by regulating the expression of inflammatory factors (Fig. 3). Zhang et al. compared SLE model mice (MRL/lpr mice) with FLI-1 heterozygote mice and found that the infiltration of inflammatory cells into the kidneys of MRL/lpr mice was significantly increased and that the level of inflammatory cell infiltration was correlated with the expression of inflammatory chemokines (CCL2, CCL3, CCL4, and CCL5) in the kidneys [59]. FLI-1 can directly bind the promoter regions of CCL2 [58] and CCL5 [59] to increase their promoter activity in a dose-dependent manner. The chemokine CXCL10 is essential for attracting inflammatory cells that express the chemokine receptor CXCR3. The downregulation of FLI-1 expression can lead to a decrease in CXCL10 mRNA levels in ECs, resulting in a decrease in the renal infiltration of CXCR3 + inflammatory cells [60]. In addition to chemokines, FLI-1 can also directly bind to the promoter region of interleukin 6 (IL-6) to activate IL-6 transcription, resulting in high expression of IL-6 in LN kidneys [61]. IL-6 significantly affects the number of renal infiltrating macrophages and lymphocytes [62]. The expression of granulocyte colony-stimulating factor (G-CSF) [63] and granulocyte macrophage colony-stimulating factor (GM-CSF) [64] in ECs is also directly regulated by FLI-1, and G-CSF and GM-CSF influence the chemotaxis of neutrophils and macrophages to the kidneys.

### The mechanisms of action of FLI-1 in the occurrence and development of PAH

PAH is a circulatory disease defined as a resting mean pulmonary artery pressure ≥ 25 mmHg, capillary wedge pressure ≤ 15 mmHg, and pulmonary vascular resistance > 3 Wood units. PAH is a highly heterogeneous disease that can be divided into idiopathic PAH and secondary PAH. Secondary PAH often occurs in SSc, SLE, viral infection, drug use, toxicity, etc. [65]. In addition to the cell types known to be involved in the pathogenesis of PAH (such as smooth muscle cells, fibroblasts and leukocytes), studies have shown that ECs activation is a key mediator of the occurrence and progression of PAH [66]. The expression of FLI-1, an important regulator of the gene network that controls endothelial homeostasis, is downregulated in PAH [67].

The role of FLI-1 in PAH mainly includes increased inflammatory gene expression and downregulated homeostatic gene expression in ECs. In the lungs of PAH patients and chronic hypoxic mice, Looney et al. reported that the expression of FLI-1 and ERG were downregulated, resulting in the significant upregulation of the expression of proinflammatory genes (CXCL10, HAS2, IL1A, CCL5, CXCL11 and VCAM1) and IFN-response genes (GBP4, IFIT2, OASL and IRF6) and the downregulation of the expression of regulatory endothelial homeostasis genes (FABP4, LYVE-1, and APLN), leading to spontaneous lung inflammation and pulmonary vascular diseases [67]. In addition, some of the previously mentioned cytokines that are regulated by FLI-1 can also affect PAH. For example, adipsin contributes to the development of PAH by activating the complement system [50], and IL-6 induces PAH by promoting cell proliferation and inhibiting apoptosis [68].

### The mechanisms of action of FLI-1 in tumorigenesis and tumour development

A tumour refers to a colony of cells that proliferate more frequently than normal tissue and in an uncontrolled way, with invasive and metastatic properties. The occurrence and development of tumours are the result of multiple hereditary and environmental factors, such as the activation of proto-oncogenes and the inactivation of tumour suppressor genes [69]. At present, the main therapeutic approaches for treating tumours are chemotherapy, radiotherapy, hormone therapy, antiangiogenic therapy and surgery. Among the different methods of tumour treatment, chemotherapy is a pivotal and common strategy because it can be used alone or in combination with other modalities (combination therapy), and it can act by impairing or targeting nutrient sources and enzymes of cancer cells and affecting hormones [70]. However, according to multiple clinical studies, drug resistance, tumour metastasis and recurrence are considered the main factors that reduce the efficacy of chemotherapy [71]. Therefore, focus begins to shift to the efficacy of antiangiogenic and targeted therapies in tumours. An increasing number of studies have shown that FLI-1 not only plays an important role in the development of a variety of tumours but also influences angiogenesis, suggesting that FLI-1 may be a good therapeutic target.

FLI-1 was initially found in mouse erythroleukaemia [5], and many studies have shown that FLI-1 is expressed not only in haematological tumours but also in Ewing’s sarcoma, breast cancer, lung cancer, bladder cancer, melanoma, lymphoma, astrocytoma, ovarian cancer, cervical cancer, colorectal cancer, nasopharyngeal carcinoma and other solid tumours [6, 72–79]. FLI-1 overexpression occurs in most tumour cells. An analysis of 4323 tumours revealed that FLI-1 was highly expressed in 46/62 Ewing’s sarcoma/primitive neuroectodermal tumours, 2/3 olfactory neuroblastomas, 7/102 small cell carcinomas of the lung, 10/34 Merkel cell carcinomas, 19/132 non-Hodgkin’s lymphomas, 9/29 medullar carcinomas of the breast, 2/3 desmoplastic small round cell tumours, and 53/74 benign and malignant vascular tumours [72]. In several breast cancer cell lines (MDA-MB231, MDA-MB436, BT-549 and HCC1395) and T- or B-lymphoblastic lymphomas, FLI-1 is highly expressed [73, 76]. Numerous studies have shown that the level of FLI-1 in tumour cells is closely related to patient prognosis. Patients with higher FLI-1 expression in ovarian cancer, astrocytoma, non-small cell lung cancer, plasmablastic lymphoma and nasopharyngeal carcinoma have poor overall survival (OS) [74–76, 79, 80]. This occurs mainly because FLI-1 regulates the expression of critical factors involved in various pathological processes, such as tumour cell growth, proliferation, differentiation, apoptosis, genomic instability, and immunity. FLI-1 promotes GATA-1 and p110 expression in leukaemia cells and inhibits the transcriptional activity of SHIP-1 and Rb by regulating the PI3K/Akt signalling pathway, thus inducing cell proliferation. Moreover, it promotes the transcriptional activity of MDM2 and Bcl-2 and inhibits the transcriptional activity of phosphorylated Bax and Bad, consequently inhibiting cell apoptosis [6].

In addition to aberrant FLI-1 expression in tumour cells, reports in the literature also indicate that FLI-1 expression is dysregulated in ECs residing in the tumour microenvironment. In tissue sections from mice with breast cancer, lung cancer and melanoma, Nagai N et al. reported that the expression level of FLI-1 in ECs in the tumour microenvironment was significantly lower than that in ECs in non-tumour microenvironments; the downregulation of FLI-1 expression was caused in part by soluble factors in the tumour microenvironment [28], including TNF-α, IL-1β, and IFN-γ.

The impact of FLI-1 on ECs within the tumour microenvironment is primarily demonstrated through its involvement in tumour metastasis, including angiogenesis and endothelium-mesenchymal transition (EndMT) processes. The EndMT process can drive the formation of cancer-associated fibroblasts, strengthen the stromal fibroblast microenvironment, reshape the vascular system, and support tumour cell proliferation and metastasis [81]. EndMT has been detected in tumour specimens from many cancer patients, including melanoma, colorectal cancer, pancreatic ductal adenocarcinoma, lung cancer, breast cancer, and glioblastoma patients [81, 82]. A wealth of evidence has highlighted the function of EndMT in tumour metastasis [81–83]. Stawski et al. downregulated FLI-1 in primary cultured human umbilical vein ECs, founded that the downregulation of FLI-1 expression mainly promotes the EndMT process by reducing the expression of VE-cadherin and inducing the morphological rearrangement of actin filaments [21]. MicroRNA-126, which is specifically expressed in ECs, is a key downstream target of the FLI-1-mediated regulation of EndMT [28].

## Summary and outlook

In conclusion, as a key transcription factor in ECs, FLI-1 promotes ECs differentiation and angiogenesis; however, the effects of FLI-1 on ECs proliferation, migration and coagulation regulation are still controversial and need further clarification. ECs differentiation promotes vascular regeneration, which is beneficial for the treatment of ischaemic cardiovascular diseases, diabetes, wound healing, stroke and other diseases [10]. More efficient ECs differentiation protocols need to be developed in the future.

**Fig. 4:**
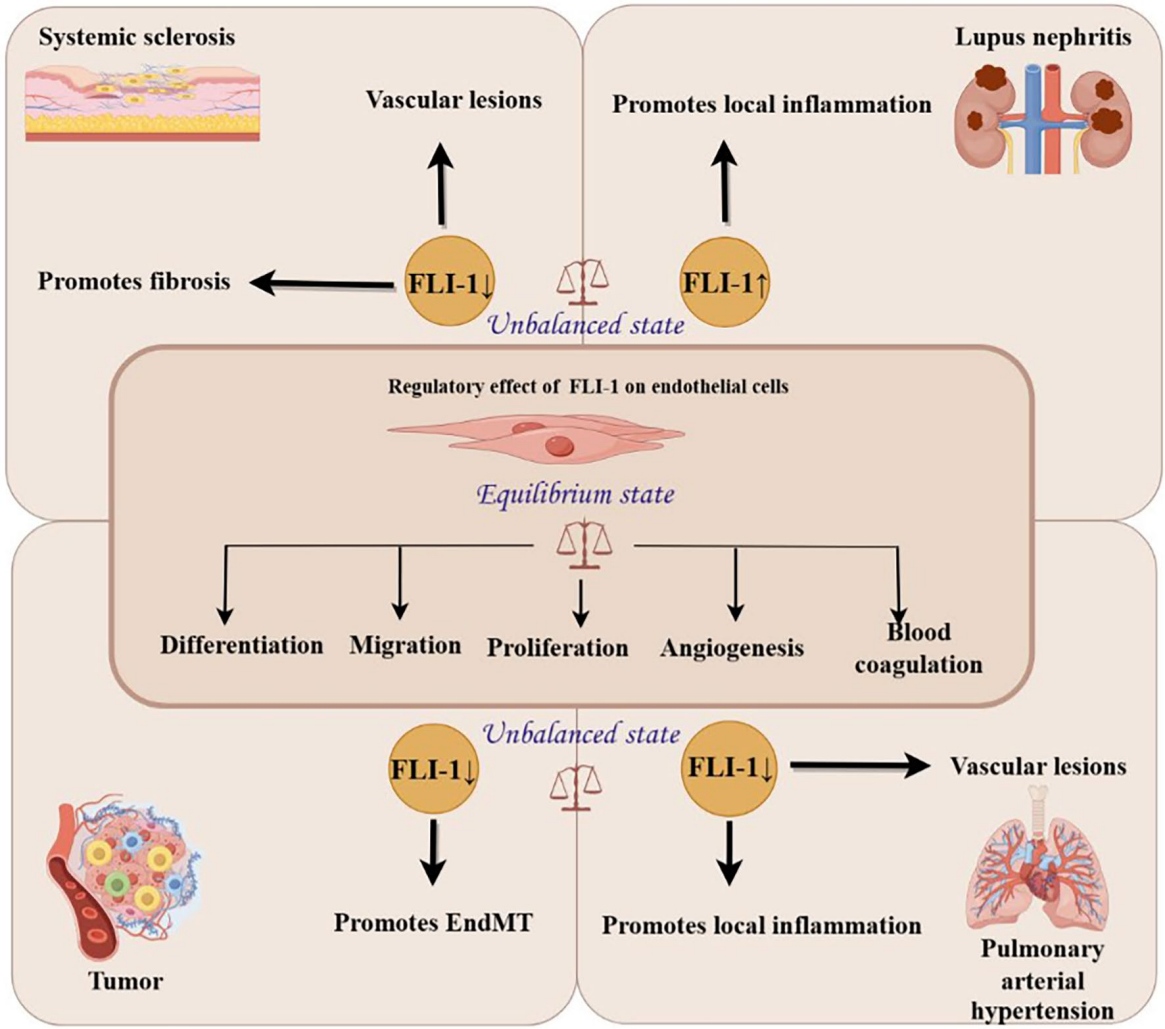
Schematic diagram showing the regulatory effects of FLI-1 on endothelial cells and diseases caused by abnormal FLI-1 expression. When FLI-1 in endothelial cells is in equilibrium, FLI-1 can regulate cell differentiation, migration, proliferation, angiogenesis and blood coagulation. When FLI-1 is unbalanced in endothelial cells, low FLI-1 expression leads to systemic sclerosis by promoting fibrosis and vascular lesions, to tumour metastasis by promoting EndMT process, and to pulmonary arterial hypertension by promoting a local inflammatory state and vascular lesions; high FLI-1 expression leads to lupus nephritis by promoting a local inflammatory state

Abnormal expression of FLI-1 in ECs may play a role in several diseases (Table 3); for example, low FLI-1 expression leads to SSc by promoting fibrosis and vascular lesions, to PAH by promoting the local inflammatory state and vascular lesions, and to tumour metastasis by promoting the EndMT process; and high FLI-1 expression leads to LN by promoting a local inflammatory state (Fig. 4).

**Table 3:** The effect of abnormal EC FLI-1 expression on human diseases

Abnormal expression of FLI-1 in ECs	Mechanism of action of FLI-1	Disease
High expression of FLI-1	Upregulates the expression of inflammatory factors	LN
Low expression of FLI-1	Upregulates the expression of profibrotic genes	SSc
	Affects angiogenesis	SSc
	Downregulates the expression of vascular homeostasis genes	PAH
	Upregulates the expression of proinflammatory genes and IFN response genes	PAH
	Facilitates the EndMT process	Tumours

*ECs* endothelial cells, *SSc* systemic sclerosis, *PAH* pulmonary arterial hypertension, *LN* lupus nephritis, *EndMT* endothelium-mesenchymal transition

Currently, FLI-1 is being used as a target for the treatment of the abovementioned diseases, and FLI-1-targeted drugs can be divided into FLI-1 activators and FLI-1 inhibitors, whose purpose is to normalize FLI-1 levels (Table 4). The mechanism of action of FLI-1 activators include (1) increasing the level of FLI-1 transcription [84]; (2) increasing the binding of FLI-1 to target gene DNA [85]. The mechanism of action of FLI-1 inhibitors is as follows: (1) decreasing the transcription of FLI-1 [86]; (2) decreasing the translation of FLI-1 [87]; (3) decreasing the binding activity of FLI-1 to target gene DNA [88]; and (4) binding FLI-1 protein and preventing FLI-1 from acting [89]. Most FLI-1-targeted drugs mainly target FLI-1 in tumour cells or fibroblasts, with little attention given to FLI-1 in ECs. Drugs targeting FLI-1 in ECs mainly include YK-4–279, an FLI-1 inhibitor used to treat diseases such as premature retinopathy and diabetic retinopathy by inhibiting FLI-1 transcription in ECs and reducing angiogenesis [90]; camptothecin, an FLI-1 inhibitor used to treat LN by inhibiting FLI-1 translation in ECs and reducing inflammatory factors in the kidney [91]; and cyclophosphamide, an FLI-1 agonist used to treat SSc by normalizing angiogenesis by promoting FLI-1 transcription in ECs [92].

**Table 4:** Mechanisms of action of existing FLI-1-targeted drugs

	FLI-1-targeted drugs	Mechanism	Targeted disease	References
FLI-1 inhibitors	Diterpenoids: A661 and A665	Decreases FLI-1 transcription	Haematologic tumours	[86]
	YK-4–279	Decreases FLI-1 transcription	Vascular proliferative disorders and tumours	[90]
	Flavagline-like compounds: A1544 and A1545	Decreases FLI-1 translation	Haematologic tumours	[87]
	Calcimycin	Decreases the binding activity of FLI-1 to target gene DNA	Haematologic tumours	[88]
	Lumefantrine	Binds FLI-1 protein	Glioblastoma multiforme	[89]
	Topoisomerase inhibitors: camptothecin and topotecan	Decreases FLI-1 translation	LN	[91]
FLI-1 activators	Ciprofloxacin	Increases FLI-1 transcription	SSc	[84]
	Cyclophosphamide	Increases FLI-1 transcription	SSc	[92]
	Phorbol ester-like compounds	Increase FLI-1 transcription	Haematologic tumours	[113]
	Bosentan	Increases the binding activity of FLI-1 to target gene DNA	SSc	[85]
	3’, 5’-diprenylated chalcone inhibitor	Increases FLI-1 transcription	Prostate cancer	[78]
	Small molecule peptide apelin	Increases FLI-1 transcription	Acute lung injury	[114]

Due to the resistance of tumours to chemotherapeutic drugs, there is an urgent need to develop more effective therapeutic agents. The abnormal expression of FLI-1 in malignant tumour cells and excessive vascular growth in the local tumour microenvironment can promote tumour occurrence and metastasis. Combined with the key role of FLI-1 in angiogenesis mentioned above, FLI-1-targeted inhibitors seem to not only inhibit the malignant proliferation of tumour cells but also constitute one of the strategies for inhibiting angiogenesis to reduce tumour metastasis.

The immune microenvironment is a highly complex structured ecosystem in which tumour cells coordinate with the tumour-supporting environment by reorganizing and reprogramming host cells. During tumorigenesis, long-term inflammatory signals, hypoxia, low pH and changes in the levels of metabolites inhibit the activity of antitumour immune cells and cytotoxic CD8 + T cells and gradually reduce the number of these cells, which exacerbates immune escape and tumour progression [93]. Studies have shown that low-dose FLI-1 inhibitors can differentially activate CD8( +) T cells, promote memory T-cell (TM) differentiation, reduce exhausted T-cell (TEX) differentiation by upregulating Runx3 and Jak1 expression [94], increase the TM-mediated antitumour immune response, and reduce TEX-mediated immune escape. Therefore, we speculate that FLI-1 inhibitors may also have synergistic antitumour effects with immunodetection site inhibitors; however, more data are needed to confirm that hypothesis.

Notably, in the tumour microenvironment, the downregulation of FLI-1 expression in ECs can promote the EndMT process and facilitate tumour metastasis. Therefore, it is necessary to assess the expression of FLI-1 in ECs while applying FLI-1 inhibitors to avoid downregulating FLI-1 expression, which causes EndMT. How to detect the expression level of FLI-1, and whether it can be indirectly reflected by detecting downstream factors of FLI-1 needs to be explored further. In ECs, FLI-1 can transcriptionally activate CCL2 [58], CCL5 [59], CXCL10 [60], IL-6 [61], G-CSF [63], GM-CSF [64], CXCL5 [42], LIF [48], CNN1 [54], NRP1 [53] and EPCR [36] and transcriptionally inhibit the expression of CXCL6 [43], CCR6 [41], AQP1[47], trappin-2 [49], LL-37 [51], adipsin [50], chemerin [26], and CTSL [52].

In addition, since FLI-1 can be expressed and play a role in blood cells, immune cells, etc., FLI-1-targeted drugs may inadvertently affect the function of other normal cells while exerting local antitumour effects. Therefore, methods for the delivery of FLI-1-targeted drugs that reduce the impact on normal cells while exerting local antitumour effects should also be a focus of future research.

Currently, there are two main strategies for delivering FLI-1-targeted drugs to the tumour microenvironment. The first strategy involves binding to receptors expressed specifically on the target cells; for example, antibody‒drug conjugates (ADCs) may improve the targeting of topoisomerase inhibitors (a kind of FLI-1 inhibitors) to tumour cells for increased effectiveness. deruxtecan is composed of an anti-HER2 antibody attached to a topoisomerase I inhibitor via an enzymatically cleavable peptide linker [95], patritumab deruxtecan is composed of an anti-HER3 antibody attached to a topoisomerase I inhibitor payload via a tetrapeptide-based cleavable linker [96], and sacituzumab govitecan is composed of an anti-Trop-2 antibody coupled to a topoisomerase inhibitor via a unique hydrolyzable linker [97]. To date, these ADCs have been successfully used to treat various types of cancer, including breast cancer, gastric cancer, and non-small cell lung cancer [95, 98–101]. Topoisomerase inhibitors can also bind to anti-B7-H4 antibody [102] and anti-B7-H3 antibody [103] to form ADCs, but these drugs are still in the clinical research stage. Another strategy for targeted medication delivery is incorporation in nanomaterials [104, 105]. Nanotechnology works through two mechanisms: active release mechanisms triggered by specific triggers within the cell or tumour microenvironment, and passive release mechanisms dependent on permeation and retention effects [106]. Liposomes, micelles composed of polymers, dendrimers composed of quantum dots, nanotubes composed of carbon, mesoporous nanoparticles composed of silica, metallic/magnetic nanoparticles, quantum dots and green synthesized nanoparticles are a few of the nanomaterials that show promise as novel cancer therapies [106–108]. Currently, tissue nanotransfection as a nanotechnology that can drive angiogenic cell therapy for nerve repair/regeneration by delivering the reprogramming factors Etv2, Foxc2, and FLI-1 [109]. A variety of nanomaterials, for example, N-(2-hydroxypropyl) methacrylamide copolymers, polyglutamic acid, polyethylene glycol, and carboxymethyldextran polyalcohol polymers, can deliver camptothecin (a kind of FLI-1 inhibitors) to tumour cells to improve its anticancer activity [110–112].

Overall, this review indicates that FLI-1 is a promising therapeutic target for SSc, LN,PAH and tumours, particularly for tumour treatment. However, there are still some problems with FLI-1-targeted drugs that need to be answered, more research focused on FLI-1-targeted drugs is needed in the future.
